# Interpersonal Behaviors Questionnaire (IBQ) applied to parenting of emerging adults: dimensional structure and criterion validity

**DOI:** 10.1186/s40359-022-00983-6

**Published:** 2022-12-02

**Authors:** Rimantas Vosylis, Rasa Erentaitė

**Affiliations:** 1 Institute of Psychology, Mykolas Romeris University, Ateities Str. 20, 08303 Vilnius, Lithuania; 2 Faculty of Social Sciences, Arts, and Humanities, Kaunas University of Technology, Kaunas, Lithuania

**Keywords:** Parenting, Interpersonal context, Basic psychological needs, Self-determination theory, Emerging adulthood

## Abstract

**Background:**

The prolonged transitions to adulthood strengthen interest in parenting characteristics that can shape emerging adults’ development and progression to full-fledged adulthood. It also strengthens interest in instruments suitable for measuring such parenting characteristics. The current study contributes to this area of research by applying the Interpersonal Behavior Questionnaire (IBQ), to assess parenting behaviors that are pertinent for emerging adults’ development and wellbeing, and seeks evidence of its dimensional structure and criterion validity.

**Method:**

The sample of the study consisted of 600 emerging adults (*M*_*age*_ = 24.94, *SD*_*age*_ = 3.03, range 19–29 years; 52.3% women). The dimensionality of IBQ was assessed by testing a sequence of theoretically plausible models representing alternative factor score structures. Criterion validity was investigated by exploring the associations between the IBQ dimensions and basic psychological need satisfaction and frustration, negative affectivity, and thriving.

**Results:**

The findings provide evidence of a hypothesized six-factor structure, but advocate for the use of exploratory structural equation as it may more accurately reflect associations between the six dimensions. Findings also provide evidence of criterion validity.

**Conclusions:**

The findings indicate that IBQ applied to parenting targets dimensions highly relevant for EAs’ flourishing or impoverished functioning. Findings also offer evidence of structure and criterion validity for the IBQ applied to emerging adults’ parent behaviors. As such, researchers may find IBQ attractive, as it is relatively concise yet also a holistic measure that captures the behaviors that both support and thwart an emerging adult’s need for autonomy, competence, and relatedness. Findings also shed light on the process of becoming an adult, the shift in parent–child relationships during this process, and emerging adults’ wellbeing.

**Supplementary Information:**

The online version contains supplementary material available at 10.1186/s40359-022-00983-6.

## Background

A transition from parental dependence to self-reliance that represents achieving adulthood [1]intensifies and finalizes during emerging adulthood (EA; the period between 19 and 25/29 years of age; [2]. During these years, relationships between emerging adults (EAs) and their parents undergo a transformation [1] that is characterized by a gradual decrease in the frequency of parent–child interactions and an increase in mutuality, reciprocity, horizontality, closeness, openness, and overall harmony in parent–child relationships [3, 4]. While this transformation is considered normative for this period of life, there is significant variability in the timing of these changes. That is, changes in parent–child relationships can occur relatively early for some EAs and much later for others [1]. This variability can partially be attributed to prolonged semi-dependence on parental financial and social support, which now characterizes many EAs, as it takes longer for recent generations of young people to reach financial self-reliance [5, 6]. As a result, parents remain active agents in the development of many EAs and can significantly affect their wellbeing [3].

The heterogeneity of adulthood transitions and prolonged EAs’ dependence on family support strengthen interest in parenting practices, which shape EAs’ development and progression to full-fledged adulthood, and theory-based instruments suitable for measuring such parenting characteristics (e.g., [7]). The current study contributes to this area of research by applying the Self-Determination Theory (SDT)-based scale of need-supportive and -thwarting interpersonal context, namely Interpersonal Behavior Questionnaire (IBQ; [8, 9]), to assess parenting behaviors that are pertinent for EAs’ development and wellbeing. While doing so, the study also addresses its dimensional structure and seeks evidence of its criterion validity.

### The role of autonomy-, competence-, and relatedness-supportive and -thwarting parenting in emerging adults’ wellbeing and maturation process

According to the SDT, a person is more likely to flourish and follow a more adaptive development trajectory when one’s interpersonal context supports three basic psychological needs (BPN)—the ones for autonomy, competence, and relatedness [10, 11]. SDT posits that the lack of support for BPN may fail to foster the growth potential of individuals, but the interpersonal context that actively blocks or *thwarts* the satisfaction of BPN may impede growth, promote ill-being and impoverished functioning [12, 13, 14]. The theory also states that the satisfaction of BPN is necessary for an individual’s growth and positive functioning at any developmental stage [14, 15]. This also applies to EA, in which attaining *autonomy* without making sacrifices in *relatedness* in relationships with parents is considered one of the central developmental tasks [1, 16, 17]. At the same time, *competence* (self-efficacy, mastery) is considered an essential resource in navigating the transition to adulthood [18, 19, 20]. More so, SDT stresses that the satisfaction of three basic psychological needs is a universal requirement for wellness and adaptive growth,it applies to everyone regardless of the sociocultural context [11].

As parents still play a significant role in EAs’ development [6], they can also *support* or *thwart* the satisfaction of three needs and shape the inherent propensities toward the positive development and wellbeing of their EA children. For example, autonomy-supportive parenting (often referred to as parental promotion of volitional functioning [21]; was linked to EAs’ higher psychological wellbeing [22], fewer internalizing problems [23, 24, 25], fewer externalizing problems, lower depression, and loneliness [26], and a better adjustment in an academic and social context [22]. In contrast, autonomy-thwarting parenting (referred to as psychological control; [21] was linked to perceived incompetence in reaching adulthood [3], dysfunctional individuation [27], higher depression symptoms, lower self-esteem [28], higher internalizing problems [23], difficulties in establishing general [29] and vocational identity commitments [3], and relational aggression [30]. Compared to other parenting combinations, the combination of low autonomy-support and high autonomy-thwarting, as shown in the person-oriented studies, was also most strongly predictive of problems in multiple domains of psychological functioning [31, 32].

Relatedness-supportive parenting was also linked with the psychological functioning of EAs. For example, both autonomy- and relatedness-supportive parenting were linked to lower externalizing problems, depression, and loneliness, while the relatedness-supportive parenting also contributed to lower levels of stress and anxiety [26]. Relatedness-supportive parenting was also linked to academic self-efficacy and individuation of EAs involved in tertiary education [33]. While fewer studies addressed the effects of competence-supportive parenting, one study has shown that among the three needs, the satisfaction of the need for competence in the relationships with parents was most strongly related to a more optimal personal identity development of EAs [34].

In sum, plenty of evidence suggests that parenting characteristics reflecting need-supportive or -thwarting behaviors may play a major role in the adaptive or maladaptive development and wellbeing of EAs. Nevertheless, the majority of studies with EAs on need-supportive parenting focus only on autonomy, and relatively few address the two remaining needs. Such a situation may be explained by a lack of validated and holistic tools that target parental behaviors related to the satisfaction and frustration of all three needs. One tool that could measure the characteristics of family context from the SDT perspective is the IBQ. It was initially developed to assess BPN satisfaction and frustration in the overall interpersonal context of an individual [8, 9], but it was also adapted to the contexts of general education [35], physical education [36], and sports [8, 9]. Being relatively flexible, IBQ can be adapted to measure parenting, which would provide a possibility to more extensively study and bring more light on the role of parenting during the years of EA.

### The current study

In the current study, we apply the IBQ to measure general parenting as perceived by EAs and seek evidence of its structure and criterion validity. The IBQ has six scales that measure six SDT-based interpersonal styles. Three of these measure BPN supportive interpersonal behaviors, and three—BPN thwarting. Table 1 provides definitions of each style.

**Table 1:** Conceptual definitions of the six need-supportive and need-thwarting interpersonal context dimensions and sample items

Dimension/scale	Definition	Sample item (My parents…)	Reliability
Autonomy support	Interpersonal behaviors characterized by the encouragement of self-initiation, provision of choices, and acknowledgment of other’s perspectives	Give me the freedom to make my own choices	ρ = .83
Competence support	Interpersonal behaviors characterized by the display of positive attitudes towards success and belief in one’s capabilities, provision of positive feedback	Encourage me to improve my skills	ρ = .81
Relatedness support	Interpersonal behaviors characterized by provision of unconditional positive regard, support, and care for others	Are interested in what I do	ρ = .70
Autonomy thwarting	Interpersonal behaviors characterized by the excessive use of control and demands without providing a rationale	Pressure me to do things their way	ρ = .82
Competence twharting	Interpersonal behaviors characterized by provision of negative feedback and discouragement from taking on complex tasks	Point out that I will likely fail	ρ = .88
Relatedness thwarting	Interpersonal behaviors characterized by emotional rejection and alienation in relationships	Do not comfort me when I am feeling low	ρ = .86

While addressing the internal structure of the measure, we also consider the debate of dimensionality that surrounds the instruments targeting BPN satisfaction/frustration and BPN supportive/thwarting contexts. This debate is focused on what set of dimensions (correlated factor, bi-factor, or second-order models) best represent or could be used to represent such measures and what measurement models (CFA-type or ESEM-type models) are best suited to capture the underlying factors. This debate stems from the numerous ways these six dimensions were modeled in the studies on BPN (for an overview, see [37, 38]). We address this issue by investigating the dimensionality of IBQ applied to parenting using an analytical framework utilized in the studies with IBQ targeting general interpersonal context [38] and the BPN satisfaction and frustration [37] that includes testing a sequence of theoretically plausible models representing alternative factor score structures.

In this framework, there is an established sequence of testing the structure of IBQ, and uniform names of the models are used to refer to specific model solutions. We follow this sequence and names in our study to facilitate a comparison of our findings to those of the previous research. Models one to seven are “correlated-factor” models, which hypothesize the presence of a different set of correlated dimensions. Specifically, model 1 (M1), a CFA-type (confirmatory factor analysis type) model, hypothesizes the presence of one global overreaching need-nurturing interpersonal context (in our case—parenting) dimension. Models 2, 4, and 6 are CFA-type models hypothesizing the presence of two (M2; need-supportive and need-thwarting interpersonal styles), three (M4; autonomy-, competence-, and relatedness-supportive style), and six (M6; autonomy-, competence-, and relatedness need-supportive and autonomy-, competence-, and relatedness need-thwarting) interpersonal styles. Model 6 represents the score structure reported in the initial IBQ validation study [8, 9] and was expected to have a better fit than Models 1, 2, and 4. Models 3, 5, and 7 are respective ESEM (exploratory structural equation model) alternatives of models 2, 4, and 6. These models have the same set of factors but are modeled using the ESEM approach, which does not constrain any cross-loadings to zero.

The next set consists of bi-factor models that, in addition to the dimensions modeled in the “correlated-factor” models (models 1 to 7), include one or two general factors. A unique contribution of bifactor models is that they enable extracting the balanced need-nurturing context as a general factor and the satisfaction or frustration of a specific need as specific factors (e.g., [38]. Models 8, 10, and 14 are bi-factor CFA alternatives of M2, M4, and M6, which, in addition to the set of specific factors, also include a single general “need-nurturing context” factor that loads on all indicators. Models 9, 11, and 15 are respective ESEM alternatives of M8, M10, and M14. Models 12 and 16 are respective CFA alternatives of M4 and M6, but both include two correlated general factors instead of one. Models 13 and 17 are ESEM alternatives of M12 and M16.

Lastly, in line with the previous study that used this framework to investigate the dimensionality of IBQ in a general interpersonal context [38], we also tested higher-order alternatives for the M14 (labeled as M14.2), M15 (labeled as M15.2), M16 (labeled as M16.2), and M17 (labeled as M17.2). The higher-order models tested a hypothesis that the shared variance between the six first-order factors is accounted by one (M14.2 and M15.2) or two (M16.2 and M17.2) superordinate higher-order dimensions. Such models are interesting as they test if the six dimensions can be summarized and represented by more general dimensions.

To obtain evidence of criterion validity, we also investigate the associations between the dimensions of need-supportive and -thwarting parenting and a set of external variables, namely BPN satisfaction and frustration, and negative affectivity and thriving. Lastly, since gender and age may also shape child and parent relationships during emerging adulthood [3], we sought evidence of generalizability of the IBQ’s factor structure and tested for measurement invariance in groups of age and gender.

## Methods

### Participants, procedures, and missing data

In the current study, we use the first wave of data from a three-wave longitudinal research project (see [39], for a more thorough description of the study). The sample includes 600 emerging adults (*M*_age_ = 24.94, *SD*_age_ = 3.03; 52.3% women). The participants were recruited from a Lithuanian online survey panel. A controlled quota sampling strategy ensured that the distribution of occupational status (educational and employment status configuration) in the sample reflected the general situation of emerging adults in Lithuania (Statistics Lithuania 2021). The participants were diverse in terms of parental socioeconomic status and other demographic characteristics. Eleven participants (1.8%) did not complete measures targeting perceptions of parenting. Consequently, the effective sample used in this study was 589 participants. Additional file 1 provide more details on participant characteristics.

The participants were recruited by sending random invitations to the panel members of a predetermined age (19–29 years old) until the established quotas were filled. Before proceeding to the survey, participants were acquainted with the purpose of the study and signed consent forms. Participants were rewarded with 1–2 Euros worth tokens for taking a survey.

## Measures

*To assess the need-supportive and -thwarting parenting*, we slightly modified the IBQ [8, 9]. The original instructions asking the participants to think about the people in their life in general were replaced by the instructions to think about one’s own parents. IBQ assesses six interpersonal behavior styles, with four items each: *autonomy-supportive* and *-thwarting, competence-supportive* and *-thwarting*,*relatedness-supportive* and *-thwarting*. The items were rated on a five-point scale (1 = Do not agree at all; 5 = Completely agree). Table 1 provides item examples and composite reliability for each scale.

*Basic psychological need satisfaction and frustration* were assessed using a scale (BNSFS) developed by Chen et al. [12]. It has six subscales, each assessed with four items: *autonomy satisfaction* (e.g., “I feel that my decisions reflect what I really want”; α = 0.78) and *frustration* (e.g., “My daily activities feel like a chain of obligations”; α = 0.74); *competence satisfaction* (e.g., “I feel capable at what I do”; α = 0.85) and *frustration* (e.g., “I feel insecure about my abilities”; α = 0.85); *relatedness satisfaction* (e.g., “I experience a warm feeling with the people I spend time with”; α = 0.87) and *frustration* (e.g., “I have the impression that people I spend time with dislike me”; α = 0.83). The participants rated the items on a five-point scale (1 = Completely untrue; 5 = Completely true).

*Negative affectivity* was assessed using the Depression, Anxiety, and Stress Scale [40]. It assesses depression, anxiety, and stress symptoms with 12 items (e.g., “I felt sad, down, and blue”) scored on a four-point scale (1 = “Did not apply to me at all”,4 = “Applied to me very much, or most of the time”). Averaged items formed a reliable (α = 0.91) negative affectivity score.

*Thriving* was measured using the Brief Inventory of Thriving [41]. The instrument includes 10 items (e.g., “My life is going well”,α = 0.93) scored on a five-point Likert-type scale (1 = “Strongly disagree”; 5 = “Strongly agree”).

All measures were translated into Lithuanian using a teamwork approach, which involved conducting multiple translations by two study contributors and one professional translator. The team discussed all translations, and the best-translated items were selected for the main study. All instruments are free to use for academic research purposes. Permission to adapt the IBQ to parenting behaviors was received from the author of the instrument.

### Data analysis

All models were estimated using the exact specification used in the earlier studies [37, 38] to compare our findings to those of the previous studies directly. All analyses were conducted using the *Mplus 8.7* software. We used the robust maximum likelihood estimator (MLR) to assess the models. Target rotation [42] was used in ESEM models. An oblique rotation strategy was used for the correlated-factor ESEM models (Models 3, 5, and 7) and orthogonal for the bi-factor models (Models 9, 11, 13, 15, and 17). Higher-order ESEM models (M15.2 and M17.2) were estimated in a two-step approach, using the ESEM-within-CFA strategy [43].

To choose the best representation of the dimensional structure, we followed the recommendations of assessing bi-factor CFA and ESEM models [43, 44, 45], which, in addition to examining the model-data fit, strongly advocate for close examination of item-factor and between factor associations. Specifically, when examining the models, we first assessed their fit with the data. To assess model fit we used RMSEA (Root Mean Square Error of Approximation) and CFI (Comparative Fit Index). RMSEA lower than 0.05, accompanied by CFI higher than 0.95, indicated good model-data fit [46]. Models that were not characterized by good fit were not further examined. Once good-fitting models were identified, we closely examined the pattern of factor loadings and between-factor correlations to identify models with a structure that matches the theoretical expectations and has a meaningful and interpretable structure.

To obtain evidence of criterion validity, we investigated the correlations between the dimension of need-supportive and need-thwarting parenting and basic psychological need satisfaction and frustration and negative affectivity and thriving. Measurement invariance (MI) analysis was performed by testing a series of models that impose equality constraints across groups for factor loadings (weak), item intercepts (strong), and residual error variances (strict) (Brown 2015). At each step, we evaluated how the model with fewer constraints compares to the model with more constraints. A certain level of invariance was supported if the constraints did not result in a substantial decrease (equal to or higher than 0.01) of CFI supplemented by a substantial (equal to or higher than 0.015) increase in RMSEA [47].

## Results

### Correlated factor models

Table 2 summarizes the fit of the seven correlated-factor models. Models 1 through 4 were not characterized by a good fit with data and were not further considered. Model 5 fit the data well, but the three factors (autonomy, competence, and relatedness) were poorly defined in terms of factor loadings. Expectedly, six-factor models 6 and 7 had a good fit with the data. Also, in both of these models, all six factors were clearly defined in terms of factor loadings. However, in the CFA model (M6), the three correlations between the need-supportive parenting dimensions were over 0.80, indicating a substantial overlap between the factors and suggesting that the three need-supportive parenting factors may not be distinguishable (Table 3 presents latent correlations). The same situation was evident for the three need-thwarting parenting dimensions. The six-factor ESEM alternative (M7), on the other hand, did not exhibit this problem, i.e., the size of the correlations between the six factors did not indicate a lack of discriminant validity. The comparison of the two models (M6 vs. M7) also suggested that ESEM had a better fit with data than the CFA alternative, advocating for the ESEM solution.

**Table 2:** Model fit statistics for the different factor analysis models of the need-supportive and -thwarting parenting

Model no	Factors	Model type	χ^2^	*df*	CFI	RMSEA [95% CI]	Compared with	ΔCFI	ΔRMSEA
M1	Nr	CFA	1818.428^***^	252	.723	.103 [.098 .107]	M6	− .241	.065
M2	S, T	CFA	859.531^***^	251	.893	.064 [.059 .069]	M6	− .071	.026
M3	S, T	ESEM	725.039^***^	229	.912	.061 [.056 .066]	M7	− .073	.029
M4	A, C, R	CFA	1666.373^***^	249	.750	.098 [.094 .103]	M6	− .214	.060
M5	A, C, R	ESEM	376.764^***^	207	.970	.037 [.031 .043]	M7	− .015	.005
M6	As, At, Cs, Ct, Rs, Rt	CFA	441.029^***^	237	.964	.038 [.033 .044]	Reference model		
M7	As, At, Cs, Ct, Rs, Rt	ESEM	233.422^***^	147	.985	.032 [.024 .039]	M6	.021	− .006
M8	Specific: S, F; General: Nr	B-CFA	557.796^***^	228	.942	.050 [.044 .055]	M2	.049	− .014
M9	Specific: S, F; General: Nr	B-ESEM	376.764^***^	207	.970	.037 [.031 .043]	M3	.058	− .024
M10	Specific: A, C, R; General: Nr	B-CFA	908.047^***^	228	.880	.071 [.066 .076]	M4	.130	− .027
M11	Specific: A, C, R; General: Nr	B-ESEM	320.065^***^	186	.976	.035 [.028 .041]	M5	.006	− .002
M12	Specific: A, C, R; General: S, T	B-CFA	428.975^***^	227	.964	.039 [.033 .044]	M4	.214	− .059
M13	Specific: A, C, R; General: S, T	B-ESEM	324.684^***^	182	.975	.036 [.030 .043]	M5	.005	− .001
M14	Specific: As, At, Cs, Ct, Rs, Rt; General: Nr	B-CFA	873.423^***^	228	.886	.069 [.064 .074]	M6	− .078	.031
M14.2	Specific: As, At, Cs, Ct, Rs, Rt; General: Nr	H-CFA	902.993^***^	246	.884	.067 [.063 .072]	M6	− .080	.029
M15	Specific: As, At, Cs, Ct, Rs, Rt; General: Nr	B-ESEM	Did not converge						
M15.2	Specific: As, At, Cs, Ct, Rs, Rt; General: Nr	H-ESEM	247.541^***^	156	.984	.032 [.024 .039]	M7	− .001	.000
M16	Specific: As, At, Cs, Ct, Rs, Rt; General: S, T	B-CFA	499.444^***^	227	.941	.045 [.040 .051]	M6	− .023	.007
M16.2_re_	Specific: As, At, Cs, Ct, Rs, Rt; General: S, T	H-CFA	550.293^***^	245	.946	.046 [.041 .051]	M6	− .018	.008
M17_re_	Specific: As, At, Cs, Ct, Rs, Rt; General: S, T	B-ESEM	151.833^*^	122	.995	.020 [.006 .030]	M7	.010	− .012
M17.2	Specific: As, At, Cs, Ct, Rs, Rt; General: S, T	H-ESEM	223.854^***^	155	.988	.027 [.019 .035]	M7	.003	− .005

Nr, need-nurturing parenting; S, need-supportive parenting style; T, need-thwarting parenting style; A, autonomy; C, competence; R, relatedness; As, autonomy-supportive parenting; Af, autonomy-thwarting parenting; Cs, competence-supportive parenting; Cf, competence-thwarting parenting; Rs, relatedness-supportive parenting; Rf, relatedness-thwarting parenting. ΔCFI and ΔRMSEA represent the change of fit indices from the previous model. re, restricted maximum likelihood estimation

***p < .001, *p < .05

**Table 3:** Internal and criterion validity correlations (N = 589)

	Dimensions of need-supportive and need-thwarting parenting							
	ASP	ATP	CSP	CTP	RSP	RTP	NSP	NTP
*Latent correlations between the six need-supportive and -thwarting parenting dimensions in the six-factor CFA model (below diagonal) and ESEM models (above diagonal)*								
Autonomy-supportive parenting (ASP)	–	− .40^*^	.57^***^	− .51^***^	.55^**^	− .44^*^	–	–
Autonomy-thwarting parenting (ATP)	− .64^***^	–	− .17	.67^**^	− .24	.46^**^	–	–
Competence-supportive parenting (CSP)	.87^***^	− .40^***^	–	− .18	.61^***^	− .34^**^	–	–
Competence-thwarting parenting (CTP)	− .69^***^	− .92^***^	− .55^***^	–	− .29	.66^***^	–	-
Relatedness-supportive parenting (RSP)	.87^***^	− .47^***^	.89^***^	− .61^***^	–	− .45^**^	–	–
Relatedness-thwarting parenting (RTP)	− .60^***^	.73^***^	− .60^***^	.82^***^	− .71^***^	–	–	–
*Correlations between the item-total scores of six need-supportive and -thwarting parenting dimensions*								
Autonomy-supportive parenting (ASP)	–						–	–
Autonomy-thwarting parenting (ATP)	− .40^***^	–					–	–
Competence-supportive parenting (CSP)	.58^***^	− .15^*^	–				–	–
Competence-thwarting parenting (CTP)	− .52^***^	.57	− .13	–			–	–
Relatedness-supportive parenting (RSP)	.54^***^	− .17^*^	.57^***^	− .22	–		–	–
Relatedness-thwarting parenting (RTP)	− .50^***^	.48^***^	− .35^***^	.67^***^	− .50^***^	–	–	–
*Correlations with the overall satisfaction and frustration of basic psychological needs*								
Autonomy satisfaction	.28^***^	− .16^*^	.32^***^	− .14	.31^***^	− .19^**^	.34^***^	− .22^***^
Autonomy frustration	− .22^***^	.28^***^	− .01	.36^***^	− .14	.29^***^	− .18^***^	.36^***^
Competence satisfaction	.33^***^	− .19^**^	.26^***^	− .22	.26^**^	− .23^***^	.33^***^	− .27^***^
Competence frustration	− .23^***^	.23^**^	− .05	.43^***^	− .14	.30^***^	− .20^***^	.38^***^
Relatedness satisfaction	.40^***^	− .30^**^	.29^***^	− .32^*^	.53^***^	− .39^***^	.46^***^	− .41^***^
Relatedness frustration	− .33^***^	.37^***^	− .17^*^	.47^***^	− .32^***^	.50^***^	− .35^***^	.52^***^
*Correlations with negative affectivity and thriving*								
Negative affectivity	− .12^**^	.30^**^	− .06	.32^**^	− .07	.28^**^	− .09^*^	.34^***^
Thriving	.39^**^	− .21^**^	.37^**^	− .29^**^	.30^**^	− .21^**^	.40^***^	− .27^***^

*p < .05; **p < .01; ***p < .001

### Bi-factor models

Table 2 summarizes the fit of the ten bi-factor models. All bi-factor CFA models (M8, M10, M12, M14, and M16) were poorly defined in terms of factor-item associations, and all, except for M12, did not have a good fit with the data. As such, bi-factor CFA models were not further considered. Two of the most complex bi-factor ESEM models, namely M15 and M17, did not converge. Using the restricted maximum likelihood estimation, i.e., forcing the residual variances to be positive, did help with convergence for M17, but not M15. The remaining bi-factor ESEM models (M9, M11, M13) converged and did have a good model-data fit. However, neither of these models was well-defined in terms of factor-item associations, and the same problem was evident for the M17. Overall, findings suggested that neither CFA nor ESEM bi-factor models were applicable to represent the structure of IBQ applied to parenting.

### Higher-order models

Table 2 summarizes the fit of the four higher-order models. The two higher-order CFA models, namely M14.2 and M16.2, did not fit the data well, and the fit of these models was much worse than that of the M6, based on which the two higher-order models were constructed. More so, the initial run for M16.2 converged in an inadmissible solution, and a restricted maximum likelihood estimation was used to deal with this issue. However, the two ESEM alternatives, namely M15.2 and M17.2, converged well and had a good fit with the data. In addition, the fit of both models was very similar to that of the M7, based on which these two were constructed. However, of the two, the M17.2, predicting the presence of two general factors instead of one, had a slightly better fit with the data. The correlation between the two general factors was also moderate (equal to − 0.42), supporting the distinctiveness of the two general factors. Overall, the factor structure analysis provided the strongest support for the six-factor ESEM model and the second-order ESEM model, predicting two higher general dimensions of need-supporting and need-thwarting parenting.

### Criterion validity

To obtain criterion validity evidence, we explored the correlations between the dimensions of parenting and our criterion validity variables: BPN satisfaction and frustration, negative affectivity, and thriving. Considering the findings of factor structure analysis, we investigated correlations between criterion variables and the six specific need-supportive and -thwarting dimensions of parenting, as well as with the two general dimensions (overall need-supportive and need-thwarting) of parenting.

The six parenting dimensions positively correlated with the corresponding dimensions of need satisfaction and frustration, providing evidence of criterion validity. In addition, general and need-specific supportive parenting dimensions were positively and moderately correlated with thriving. Similarly, general and need-specific thwarting parenting dimensions were positively and moderately correlated with negative affectivity, further evidence of criterion validity. Table 3 presents these correlations.

### Measurement invariance across groups based on gender and age

Lastly, we proceeded to test for MI of the six-factor ESEM model. Constraining factor loadings, intercepts, and residuals did not substantially decrease the model fit. As such, results indicated that the scores obtained by the IBQ scale adapted for parenting are measurement invariant across groups based on gender. MI results are reported in Table 4.

**Table 4:** Results of measurement invariance analysis across groups based on gender (N = 589)

Model tested (model compared with)	Model fit statistics					Model comparison	
	*χ*^*2*^	*df*	CFI	RMSEA [90% CI]	SRMR	ΔCFI	ΔRMSEA
Configural	419.46***	294	.978	.038 [.029 .046]	.019	–	–
Weak (configural)	543.86***	402	.976	.035 [.027 .042]	.034	− .002	− .003
Strong (weak)	568.63***	420	.974	.035 [.027 .042]	.035	− .002	.000
Strict (strong)	609.78***	444	.971	.036 [.028 .042]	.042	− .003	.001

*χ*, Model Chi-square statistic; *df*, degrees of freedom; CFI, Comparative Fit Index; RMSEA, Root Mean Square Error of Approximation; SRMR, Standardized Root Mean Square Residual; CI, Confidence Interval

***p < .001; **p < .01

## Discussion

The primary goal of the current study was to investigate if the IBQ, a measure developed to assess the characteristics of the overall interpersonal context of an individual, can also be used to measure parenting characteristics as perceived by EA children. To do so, we investigated the structure of the scale, its criterion validity, and its measurement invariance across groups based on gender and age.

Factor analysis results for the correlated-factor models predicting one, two, or three factors in IBQ scores closely replicated the findings of two earlier investigations on IBQ structure in a general interpersonal context [37, 38], i.e., neither of these models were well-defined in terms of factor-item associations, and most had a poor fit with the data. This finding indicated that oversimplified internal structure representations, predicting one, two, or three factors, misrepresent the internal associations of the IBQ. The six-factor CFA representation of the IBQ, on the other hand, had a good fit with data, similar to that reported in the initial IBQ validation study [8, 9] and two subsequent applications to specific interpersonal contexts [8, 9, 36]. Overall, this finding indicated that IBQ applied to parental behaviors retains its hypothesized factor structure.

Notably, while the six-factor CFA model fit well with the data, the correlations between the three scales of need-supportive parenting and those between the scales of need-thwarting were very high (over 0.80), putting the discriminant validity of the scales in question. However, the six-factor ESEM model had an even better fit with data than the CFA one and substantially weaker correlations between latent dimensions. Such a finding signals that high correlations observed in the CFA model can be attributed to small cross-loadings that are present yet constrained to zero in the CFA model [45]. Since constrained to zero cross-loadings may inflate and mispresent the associations between the factors, findings suggest that the ESEM representation is more suitable for research, i.e., it may more accurately reflect associations between the six dimensions.

The results for bi-factor models for the IBQ applied to parenting behaviors were also closely in line with two earlier investigations [37, 38] and warned against using such models. CFA model predicting six specific dimensions and one general, also poorly fit with data, echoing results of earlier studies. However, deviating from two earlier investigations, an ESEM representation of the same model did not converge, which was an unexpected finding, considering that this model was advocated in two earlier studies [37, 38]. The finding could be attributed to the fact that earlier studies assessed a very generalized view of interpersonal relationships without considering a particular social context, in which the distinction between supportive and thwarting interpersonal behaviors may have been less substantial.

In contrast, the results of our study supported higher-order ESEM models. Of the tested two, a better fit was observed for the one predicting two general factors (general need-supportive parenting and general need-thwarting parenting). This finding suggests that for the analytic parsimony, a broader need-supportive parenting dimension can be used to represent autonomy-, competence-, and relatedness-supportive parenting styles, and the same principle is applicable with need-thwarting parenting. The strength of the correlation between the two general dimensions also suggested that the two general dimensions were rather distinctive and provided evidence for the discriminant validity.

Proving evidence that the six SDT-based dimensions of parenting align well with the theoretical framework proposed by the SDT, correlations between the scores of six need-specific and two general parenting dimensions were substantially associated with the dimensions of need satisfaction and frustration. This suggests that emerging adults’ sense of autonomy, competence, and relatedness is still closely related to how they see their parents behaviors. Noteworthy, all six parenting dimensions had slightly stronger associations with satisfaction and frustration of the need for relatedness. These findings suggest that parental need-supportive and -thwarting behaviors may have a stronger effect on relatedness need-satisfaction and frustration than on satisfaction/frustration of autonomy and competence needs.

Lastly, providing evidence of criterion validity, all six parenting dimensions were significantly associated with well- and ill-being. In particular, thriving was significantly positively and more strongly than negative affectivity associated with each specific need-supportive and general need-supportive parenting dimension. In the meantime, negative affectivity was significantly positively and more strongly than thriving associated with each specific need-thwarting and general need-thwarting parenting dimension. This finding closely supported the central tenets of the SDT, postulating that when one’s interpersonal contexts support the three BPN, a person is more likely to flourish [10, 11], and when interpersonal context actively blocks or thwarts BPN, a person is likely to display more impoverished functioning [12, 13, 14].

Overall, the findings indicate that IBQ applied to study parenting characteristics targets the aspects of parental interpersonal style that: (a) align well with the theoretical framework proposed by the SDT; (b) are highly relevant for EAs’ wellbeing. The former provides further support for IBQ authors’ earlier suggestions [8, 9] that IBQ is a versatile tool and may be adapted to assess the interpersonal contexts embedded in different life domains. Perhaps the essential feature of the IBQ that researchers may find attractive is that it is relatively concise yet also a holistic measure that captures the behaviors that both support and thwart an individual’s need for autonomy, competence, and relatedness. Since need-supportive and -thwarting behaviors represent distinctive dimensions that have differential implications for those facing such interpersonal styles (e.g., [48], the IBQ, when used to assess parenting, may offer a possibility to distinguish parents that engage in neither of such behaviors (e.g., indifferent), some such behaviors (e.g., only need-thwarting behaviors), or even both of these (e.g., overinvolved). While distinguishing between behaviors affecting specific needs may require the ESEM approach, the use of such an instrument may open a new research avenue for those interested in the role of parenting on emerging adults’ positive and problematic development patterns. Specifically, it may shed more light on the parental behavior patterns that may be more or less beneficial in becoming an independent, self-sufficient, and responsible person.

## Limitations

One of the study’s limitations is that EAs reported their subjective perspective on their parents’ behaviors, which may give a biased view of parenting. Nevertheless, subjective perceptions of parenting may be more important for developmental outcomes than actual parental behaviors. Also, the findings may be less generalizable to other developmental stages and other interpersonal contexts. Future studies using parents and perhaps multi-informant assessment strategies could provide valuable new evidence on the dimensionality and role of need-supportive and -thwarting parenting for EAs. Another considerable limitation of the study is that EAs reported their perspectives about parents in general, instead of reporting separately on mother’s and father’s interpersonal styles. Future studies with separate assessments are necessary to understand if measurement structures are similar for mothers’ and fathers’ interpersonal styles.

## Conclusions

Parents still play a significant role in EA children’s lives, and parenting that supports or thwarts basic psychological needs can shape their offspring’s development and wellbeing. Studies investigating the effects of need-supportive and -thwarting parenting focus on strategies that target a specific need, and very few take a broader SDT-based view and address those that support or thwart all three needs. To address a lack of comprehensive measures that assess parenting styles related to all three BPN, the study offers evidence of structure and criterion validity for the IBQ applied to EAs’ parent behaviors. It also sheds light on the process of becoming an adult, the shift in parent–child relationships during this process, and emerging adults’ wellbeing.

## Supplementary Information

**Additional file 1**. A document provides a detailed description of the characteristics of study participants and characteristics of study participants’ parental socioeconomic status.

## Data Availability

The datasets generated and/or analyzed during the current study are available in the OSF repository, https://bit.ly/3AJ0CRM (link is anonymized).

## Abbreviations

EA: Emerging adulthood
EAs: Emerging adults
SDT: Self-determination theory
IBQ: Interpersonal Behavior Questionnaire
BPN: Basic psychological needs
CFA: Confirmatory factor analysis
ESEM: Exploratory structural equation modeling
