# Supplementary material for: Interpersonal Behaviors Questionnaire (IBQ) applied to parenting of emerging adults: dimensional structure and criterion validity
Source: BMC Psychol. 2022 Dec 2;10:285. doi: 10.1186/s40359-022-00983-6 (PMC9717410; doi:10.1186/s40359-022-00983-6)
Supplement: Supplementary file 1 — Additional file 1. A document provides a detailed description of the characteristics of study participants and characteristics of study participants’ parental socioeconomic status. [file 40359_2022_983_MOESM1_ESM.docx]

**Supplementary Online Materials for**

**Interpersonal Behaviors Questionnaire (IBQ) Applied to Parenting of Emerging Adults: Dimensional Structure and Criterion Validity**

Table of Contents

[Detailed description of characteristics of study participants 3](#_Toc110331907)

[Characteristics of study participants’ parental socioeconomic status 4](#_Toc110331908)

# A detailed description of the characteristics of study participants

The current study uses the first wave of data from an ongoing three-wave longitudinal investigation conducted as part of a larger research project. For the initial assessment, 600 Lithuanian emerging adults (*M*_age_ = 24.94, *SD*_age_ = 3.03, range 19–29 years; 52.3% women) were recruited from an online survey panel, using a controlled quota sampling strategy. In line with the quotas established before the study: (a) precisely 300 participants were 19-24-year-olds and 300 were 25-29-year-olds; (b) the overall distribution of participants in occupational statuses was identical to the Lithuanian emerging adult population: 10.2% of participants were studying but were unemployed, 19.3% were studying and were employed, 12.7% were neither studying nor employed, and 57.8% were employed but not studying.

Around a quarter (22.3%) of participants were living with their parents. Around a third of participants (37.0%) indicated that their highest education was secondary school, 21.0% had a college diploma, 28.3% had a bachelor’s diploma, and 13.7% had a university master’s diploma. Most participants (87.3%) did not have a child. The majority of participants (87.3%) did not have a child, while the rest had one or more children. Around a quarter of participants (27.0%) were single, 16.2% were involved in a romantic relationship (but were not living with their romantic partner), 38.3% were living with their romantic partner, 17.2% - were married, and 1.4% chose the “other” option.

# Characteristics of study participants’ parental socioeconomic status

| **OSM Table 1.**  *Frequencies of Parental Socioeconomic Status Indicators*. | | | |
| --- | --- | --- | --- |
| **Indicator** | **Response option** | Count | % of the sample |
| What is the highest level of schooling completed by your father? | *University degree* | 134 | 22.3% |
|  | *College degree or similar* | 152 | 25.3% |
|  | *Professional school* | 171 | 28.5% |
|  | *Secondary school* | 58 | 9.7% |
|  | *Primary school* | 21 | 3.5% |
|  | *Did not finish primary school* | 5 | .8% |
|  | *I don’t know* | 59 | 9.8% |
| What is the highest level of schooling completed by your mother? | *University degree* | 185 | 30.8% |
|  | *College degree or similar* | 187 | 31.2% |
|  | *Professional school* | 130 | 21.7% |
|  | *Secondary school* | 51 | 8.5% |
|  | *Primary school* | 17 | 2.8% |
|  | *Did not finish primary school* | 2 | .3% |
|  | *I don’t know* | 28 | 4.7% |
| Is your father currently employed? | *Yes, is employed full-time* | 388 | 64.7% |
|  | *Yes, works part-time jobs* | 20 | 3.3% |
|  | *Unemployed, looking for work* | 11 | 1.8% |
|  | *Unemployed, retired* | 38 | 6.3% |
|  | *Unemployed, other reasons* | 34 | 5.7% |
|  | *Other* | 25 | 4.2% |
|  | *I can't or don't want to say* | 32 | 5.3% |
|  | *This question does not apply to me* | 52 | 8.7% |
| Is your mother currently employed? | *Yes, is employed full-time* | 445 | 74.2% |
|  | *Yes, works part-time jobs* | 12 | 2.0% |
|  | *Unemployed, looking for work* | 27 | 4.5% |
|  | *Unemployed, retired* | 31 | 5.2% |
|  | *Unemployed, other reasons* | 49 | 8.2% |
|  | *Other* | 9 | 1.5% |
|  | *I can't or don't want to say* | 13 | 2.2% |
|  | *This question does not apply to me* | 14 | 2.3% |
